# The Anticarcinogenicity Effects of Piperine and 4,5-Dihydroxypiperine in *Drosophila melanogaster* Somatic Cells

**DOI:** 10.1021/acsomega.5c13279

**Published:** 2026-03-30

**Authors:** Wellington S. Aguiar, Isabelle V. A. Pires, Lívia M. L. Barros, Pedro M. Almeida, José L. S. Sá, Benedito S. Lima-Neto, Francielle A. Martins

**Affiliations:** † Graduate Program in Chemistry, State University of Piauí, Teresina, 64002-150 Piauí, Brazil; ‡ Genetics Laboratory, Center of Natural Sciences, State University of Piauí, Teresina, 64002-150 Piauí, Brazil; § Institute of Chemistry of São Carlos, University of São Paulo, São Carlos, 13560-970 SP, Brazil

## Abstract

Asymmetric dihydroxylation of piperine (PIP) was investigated under various reaction conditions. Using AD-mix-α in the presence of methanesulfonamide, the reaction yield was approximately 51% at 25 °C, whereas the reactions with RuCl_3_/NaIO_4_ were less efficient. The product was purified, and its structure was determined using ^1^H and ^13^C NMR spectroscopy. The carcinogenicity and anticarcinogenicity of PIP and 4,5-dihydroxypiperine (dhPIP) were evaluated using the Epithelial Tumor Test (ETT) in *Drosophila melanogaster* and no carcinogenic activity was observed in the presence of PIP or dhPIP at 60, 120, 180, or 360 μM. Co-treatment with doxorubicin (0.4 mM) and either PIP (180 μM) or dhPIP (60 μM) inhibited tumor occurrence by 81% and 83%, respectively.

## Graphical abstract

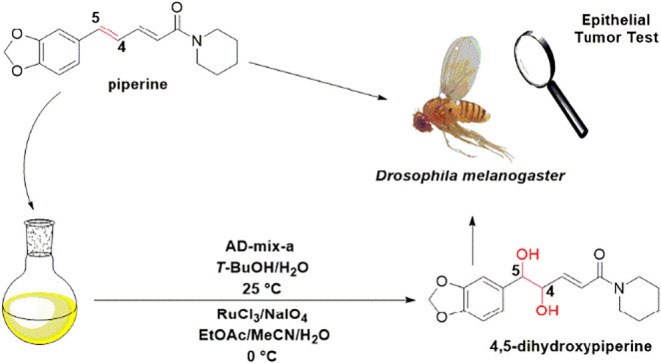

## Introduction

Chemical modification of bioactive natural products to improve their physicochemical and biological properties is an important strategy for developing new compounds for pharmacological applications.^1^ Piperine (PIP), an amide alkaloid abundantly found in the grains of *Piper nigrum* *L*., is easily isolated, and its functional groups allow for various chemical modifications (Figure 1).^2^

**Figure 1:**
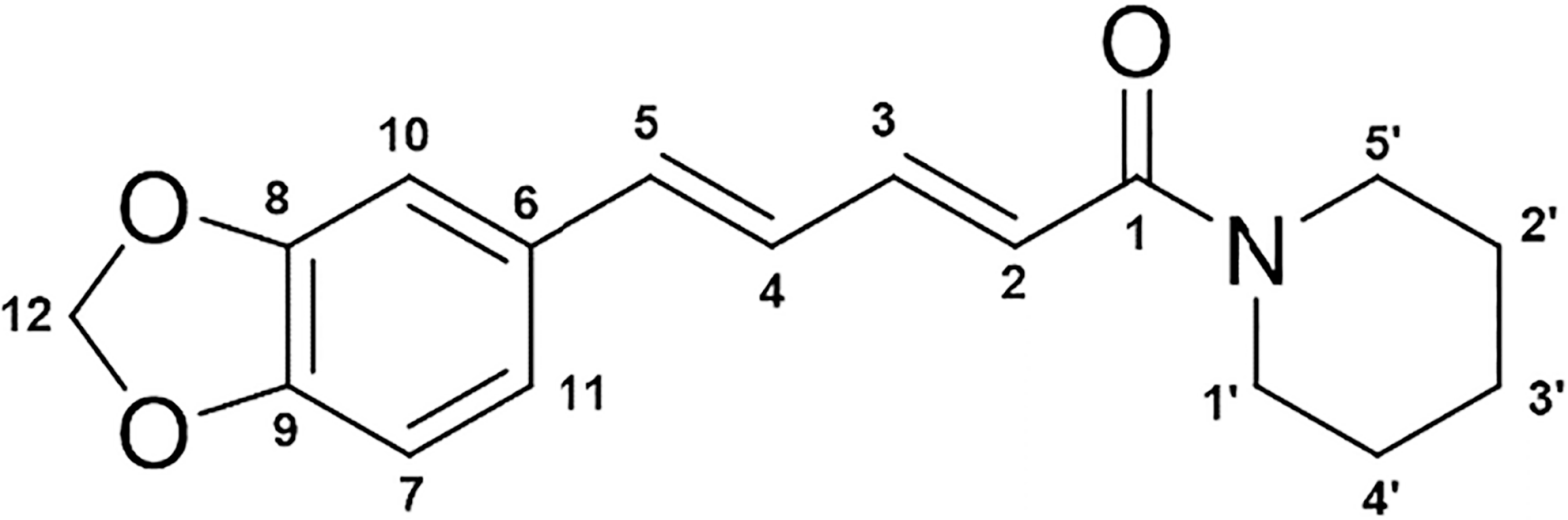
Chemical structure of piperine.

The reported activities of PIP include antioxidant, anti-inflammatory, antitumor, and antimicrobial activities,^3–6^ as well as potential applications in the treatment of Chagas disease, leishmaniasis, neurodegenerative and neurological diseases.^7–9^

Antitumor activity of PIP has been investigated due to its ability to induce apoptosis in various tumor cell lines.^10–13^ One metabolic mechanism associated with this activity is an increase in reactive oxygen species. As a result, key biomolecules essential for cell cycle progression, such as cyclins D1, B1, and A, are inhibited, leading to cell-cycle arrest.^14^ In addition, PIP activates positive regulators of apoptosis, such as caspase-3.^15^

Despite the important activities reported for PIP, its low water solubility limits its pharmacological applications. Therefore, chemical modifications aimed at minimizing or eliminating this drawback, as well as enhancing its biological activity, are of considerable interest.^16,17^

Several PIP derivatives have been reported in the literature.^18–22^ Among these, asymmetric hydroxylation of the olefin and hydroxylation of the piperidine moiety have already been described.^23,24^ It is noteworthy that the *cis*-diol group is present in the structures of several biomolecules, such as galactose, and its reactivity plays an essential role in many vital biological processes.^25^ Therefore, optimizing the protocol for asymmetric hydroxylation to obtain the diol compound using an established commercial oxidizing mixture, known as AD-mix, along with its complete characterization by NMR, is necessary.

Asymmetric dihydroxylation of olefins is usually catalyzed by osmium- or ruthenium-based compounds. A commercial mixture known as AD-mix is widely and successfully applied in the synthesis of various bioactive products, including the antiviral drugs cidofovir and buciclovir and the antitumor agents taxol and camptothecin.^26–28^ AD-mix contains potassium osmate dihydrate (K_2_OsO_2_(OH)_4_) as the catalytic oxidant, potassium ferricyanide (K_3_Fe­(CN)_6_) as the stoichiometric oxidant, and K_2_CO_3_ as an additive that facilitates the hydrolysis step. In addition, it includes a chiral ligand, a cinchona-based alkaloid, that provides high enantioselectivity and high yields for a wide range of substrates.^29^

Alternatively, dihydroxylation of olefins can be carried out under acidic conditions using ruthenium tetroxide (RuO_4_).^30^ This process was further improved by the addition of a Brønsted acid (H_2_SO_4_) or a Lewis acid (CeCl_3_).^31^ Using RuCl_3_ as a precatalyst in the presence of these acids and NaIO_4_ as a stoichiometric oxidant, in an EtOAc/ACN/H_2_O solvent mixture at 0–5 °C, only 0.25 mol % catalyst is required. This makes RuO_4_-based processes widely used in organic synthesis and applicable to a series of structurally diverse olefins.^32,33^

Considering the various studies on the interaction of PIP with DNA, the present study aimed to improve the dihydroxylation process of PIP to obtain a derivative that maintains most of its original structure and preserves its biological activity. To this end, reactions mediated by AD-mix-α and RuCl_3_/NaIO_4_ were applied to obtain the racemic diols and to investigate the carcinogenic and anticarcinogenic potentials of the resulting molecules in terms of genetic damage in aqueous medium. For this purpose, the Epithelial Tumor Test (ETT) in somatic cells of *D. melanogaster* was used as the bioassay.

## Results and Discussion

### Synthesis of Dihydroxylation from PIP

The dihydroxylation reaction of PIP catalyzed by AD-mix-α in the presence of methanesulfonamide (MSA) was studied under different conditions (Table 1). The formation of 4,5-dihydroxypiperine (dhPIP) was confirmed by ^1^H NMR measurements. Increased yields were observed, reaching up to 37% with 0.4 mol % catalytic oxidant and 1 equiv of MSA at 20 °C (Entries 1–2). Performing the reaction at 25 °C resulted in a yield of 51% (Entry 3). At 25 °C, increasing the amount of oxidant to 1 mol % produced an average yield of 95%, either with or without MSA (Entries 4–5). This result is superior to that previously reported in the literature (Entry 6).^23^ In conclusion, the reaction is sensitive to both the amount of oxidant and the temperature.

**Table 1:** Yields of 4,5-Dihydroxypiperine (dhPIP) Obtained with AD-mix-α in *t*-BuOH/H_2_O (1:1) for 24 h, under Varying Temperatures and Different Amounts of Oxidant and Methanesulfonamide (MSA)

entry	temperature (°C)	oxidant (mol %)	MSA	yields (%)
1	20	0.4		28
2	20	0.4	1 eq	37
3	25	0.4	1 eq	51
4	25	1	1 eq	96
5	25	1		94
6 ^23^	25	0.5		44

The increase in yield is believed to be directly related to the acceleration of the hydrolysis rate of the intermediate osmate ester by MSA, which may be the rate-determining step of the process. Because the substituent groups of the PIP olefin are nonpolar, hydrolysis of the osmate ester in the aqueous phase is limited. In this context, due to its cosolvent effect, MSA facilitates the transport of OH^–^ ions from the aqueous phase to the organic phase. In addition, the conjugated aromatic group in PIP, acting as an electron-withdrawing group, contributes to the formation of the osmate ester, allowing protonation by MSA, which can also act as a weak acid. This facilitates hydrolysis, in accordance with systematic studies on the effect of this additive on asymmetric dihydroxylation (Figure 2).^34^ However, a large amount of oxidant can drive the reaction directly (Entry 5).

**Figure 2:**
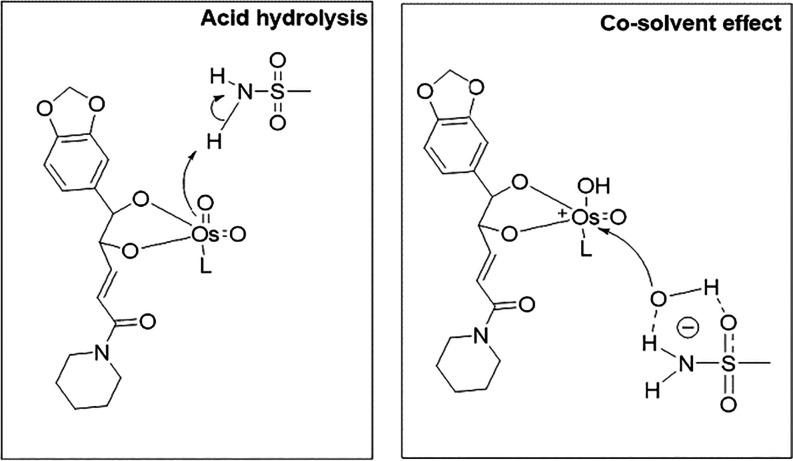
Schematic representation of the effects of acid hydrolysis and the methanesulfonamide (MSA) cosolvent on the osmate ester intermediate during the asymmetric dihydroxylation of piperine using AD-mix-α.

It is important to note that conducting the process at lower temperatures appears to prevent the loss of selectivity and increase the enantiomeric excess of the product.^35^ At this stage, MSA demonstrated a significant effect (Entries 1–2), although the yield was reduced.

Syntheses carried out at 15 °C for 24 h with increased catalyst and additive loads did not produce a sufficient amount of product to allow isolation (Table S1). Despite this, product formation was detected by mass spectrometry (Figure S1). Different ions resulting from the association of the reaction product with H^+^ (*m*/*z* = 320) and Na^+^ (*m*/*z* = 342), as well as ions corresponding to the loss of water (*m*/*z* = 302), were identified. When comparing these conditions, temperature remains the most important variable in the system, indicating that 25 °C is optimal for the asymmetric dihydroxylation of PIP using commercial AD-mix-α. However, after 7 days of reaction at 15 °C with 0.4 mol % oxidant and 3 equiv of MSA, 74% of the product was obtained. The reaction was monitored by thin-layer chromatography every 12 h until a significant decrease in the starting material was observed. The product was purified using a silica gel column with chloroform and methanol as eluents in increasing order of polarity, resulting in a white solid. After purification, the product was characterized by ^1^H and ^13^C NMR (Figures 3 and 4). All hydrogen and carbon atoms were identified, consistent with the literature data.^23^ In the ^1^H NMR spectrum (Figure 3; Table S2), the signals of the aromatic hydrogen atoms (7, 10, and 11), the olefinic hydrogens (2 and 3), the saturated methylene hydrogens of the methylenedioic ring (12), the saturated carbon-bound hydrogens attached to hydroxyl groups (4 and 5), and the saturated hydrogen atoms of the piperidine ring (1′, 2′, 3′, 4′, and 5′) were identified. The hydrogen at position 11 (δ 6.81, 1H, dd, J_ortho_ = 8.0; J_meta_ = 1.6 Hz) couples with H-7 (δ 6.76, 1H, d, J_ortho_ = 7.9 Hz) and H-10 (δ 6.88, 1H, d, J_meta_ = 1.6 Hz). The hydrogen at position 3 (δ 6.52, 1H, dd, J_trans_ = 15.3; J_3_ = 4.9 Hz) couples with H-2 (δ 6.43, 1H, dd, J_trans_ = 15.4; J_4_ = 1.4 Hz) and H-4 (δ 4.32, 1H, ddd, J = 6.6; 4.9; 1.5 Hz). The hydrogen at position 2 also couples with H-4, and H-4 also couples with H-5 (δ 4.45, 1H, d, J = 6.7 Hz). The hydrogens at position 12 (δ 5.92, 2H, dd) appear as a doublet due to magnetic nonequivalence induced by cis-dihydroxylation. The signals at positions 5′ (δ 3.53, 2H, m) and 1′ (δ 3.41, 2H, t) correspond to the methylene hydrogens directly bonded to nitrogen, whereas the signals at positions 3′ (δ 1.67, 2H, m), 2′ and 4′ (δ 1.52, 4H, m) correspond to the remaining methylene groups of the piperidine ring.

**Figure 3:**
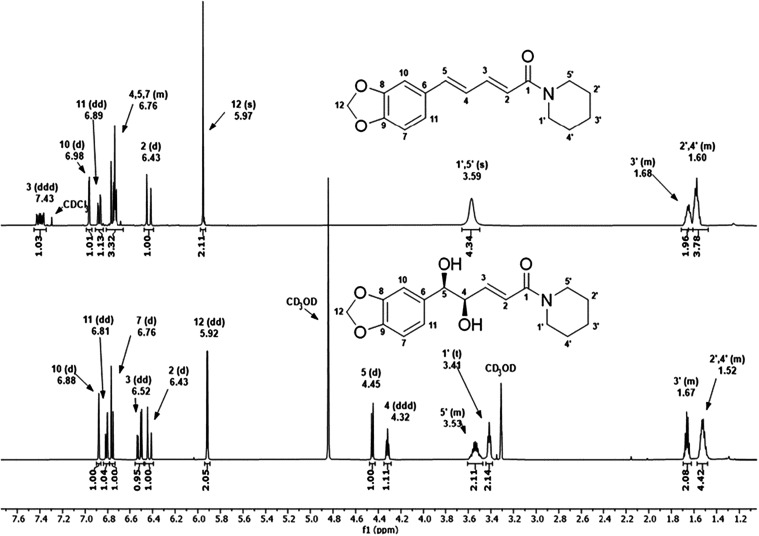
^1^H NMR spectra of PIP in CDCl_3_ and dhPIP in CD_3_OD, the latter obtained by AD-mix-α in *t*-BuOH/H_2_O (1:1) under the conditions described in entry 8 (Table S1).

**Figure 4:**
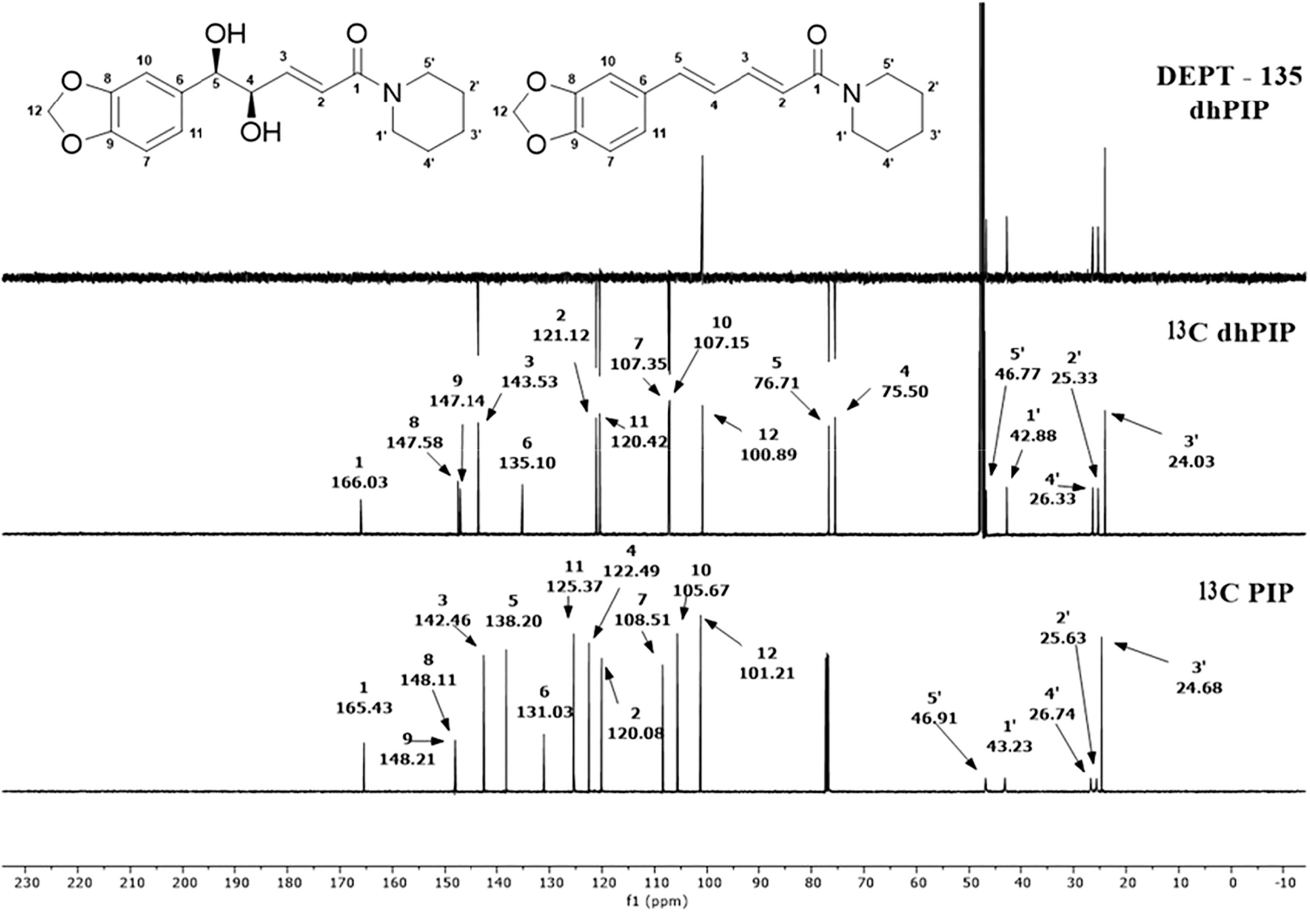
^13^C NMR spectrum of PIP in CDCl_3_ and ^13^C NMR and DEPT-135 spectra of dhPIP in CD_3_OD, the latter obtained by AD-mix-α in *t*-BuOH/H_2_O (1:1) under the conditions described in entry 8 (Table S1).

The ^13^C and DEPT-135 NMR spectra allowed the identification of all carbon types in the molecule (Figure 4). Non-hydrogenated carbons (C) did not appear in the DEPT-135 spectrum (C-1 δ 166.03, C-6 δ 135.10, C-8 δ 147.58, and C-9 δ 147.14), monohydrogenated carbons (CH) inverted their phase and pointed downward (C-2 δ 121.12, C-3 δ 143.51, C-4 δ 75.50, C-5 δ 76.71, C-7 δ 107.35, C-10 δ 107.15, and C-11 δ 120.42), and dihydrogenated carbons (CH_2_) appeared in the same phase as in the ^13^C NMR spectrum and pointed upward (C-12 δ 100.89, C-1′ δ 42.88, C-2′ δ 25.33, C-3′ δ 24.03, C-4′ δ 26.33, and C-5′ δ 46.77). Due to the similarity in chemical environments, it was not possible to differentiate C-8 and C-9, C-1′ and C-5′, or C-2′ and C-4′. On the other hand, the monohydrogenated carbons (CH) were unambiguously assigned using HSQC analysis (Figure 5), taking into account the corresponding hydrogens identified by ^1^H NMR coupling constants (J values). High enantioselectivity was obtained for the reaction (98.9% ee), as determined by normal-phase chiral HPLC analysis (Table S3, Figure S2). In addition, the circular dichroism spectrum (Figure S3) exhibited negative Cotton effects at 206 (−31.84), 214 (−23.64), and 282 nm (−5.10), consistent with the 4*R*,5*R* enantiomer, in accordance with previously reported literature data.^23^

**Figure 5:**
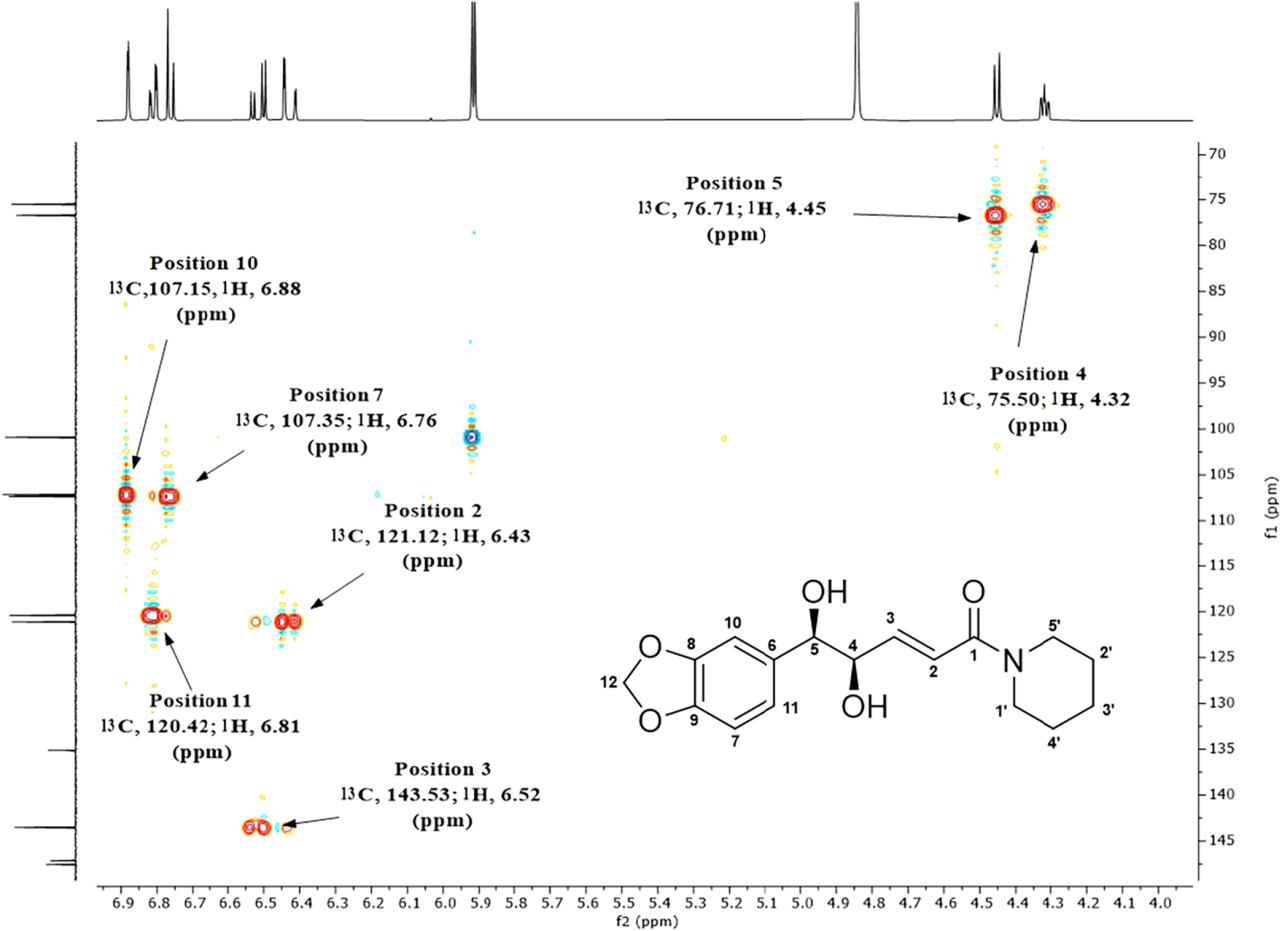
HSQC spectrum in CD_3_OD of dhPIP obtained from AD-mix-α in *t*-BuOH/H_2_O (1:1) under the conditions described in entry 8 (Table S1).

Dihydroxylation of PIP mediated by RuCl_3_/NaIO_4_ was evaluated under different conditions as an alternative protocol (Table 2). dhPIP was not produced in the absence of an additive and in an acetone/H_2_O medium (Entry 1; Figure S4). Changing the solvent to EtOAc/ACN/H_2_O resulted in the detection of dhPIP by qNMR measurements (Entries 2–4; Figure S4). However, the reaction product was not observed in the presence of a Brønsted acid, likely due to protolytic reactions involving the amide group, which is acid-labile (Entries 5–7; Figure S5).^30^

**Table 2:** Yield/Purity (*P*, Mass Fraction) of 4,5-Dihydroxypiperine (dhPIP) Determined by qNMR Analysis from the *Cis*-Dihydroxylation of Piperine Mediated by RuCl_3_/NaIO_4_ under Different Reaction Conditions at 0 °C

entry	RuCl_3_ (mol %)	NaIO_4_ (eq)	additive	solvent	time (min)	*P*dhPIP (μg/mg)
1	1	1.5		acetona/H_2_O	60	0
2	1	1.5		EtOAc/ACN/H_2_O	2	0.87
3	1	1.5		EtOAc/ACN/H_2_O	20	0.49
4	20	3		EtOAc/ACN/H_2_O	120	0.36
5	0.5	1.5	H_2_SO_4_ 20%	EtOAc/ACN/H_2_O	10	0
6	0.5	1.5	H_2_SO_4_ 20%	CH_2_Cl_2_/ACN/H_2_O	10	0
7	0.5	1.5	H_2_SO_4_ 20%	acetone/ACN/H_2_O	10	0
8	0.5	1.5	FeCl_2_ 10%	EtOAc/ACN/H_2_O	30	1.0
9	0.5	1.5	ZnCl_2_ 10%	EtOAc/ACN/H_2_O	30	0.11
10	0.5	1.5	Ce_2_(SO_4_)_3_ 10%	EtOAc/ACN/H_2_O	30	0

Protonation of the amide nitrogen can occur when the dihydroxylation reaction proceeds slowly. Problems related to protolytic reactions and highly substituted electron-poor olefins were overcome by the inclusion of Lewis acids, as confirmed by ^1^H NMR measurements (Entries 8–9, Figure 6).^31^ It is also important to note the presence of olefinic hydrogen signals from PIP at 7.33 ppm (H-3) and 6.43 ppm (H-2). Conversely, these signals were absent in Entry 10 (6.43–7.43 ppm, Figure 6), where only aromatic hydrogen signals (H-7, 10, and 11) were observed, indicating complete substrate conversion to secondary products. Several singlets in the 9.5–10 ppm region suggest the formation of aldehydes, a common outcome of this method, particularly when the resulting aldehydes are stabilized by resonance, as in the case of PIP. The spectrum from the solution in Entry 10 exhibited only two singlets in the aldehyde region, suggesting a selective reaction at one of the unsaturated positions. Reducing the amount of Lewis acid may provide a way to obtain dhPIP under the conditions of Entry 10, even if it requires a longer reaction time. This approach offers greater control and almost completely suppresses the fragmentation pathway leading to aldehyde formation.^31^

**Figure 6:**
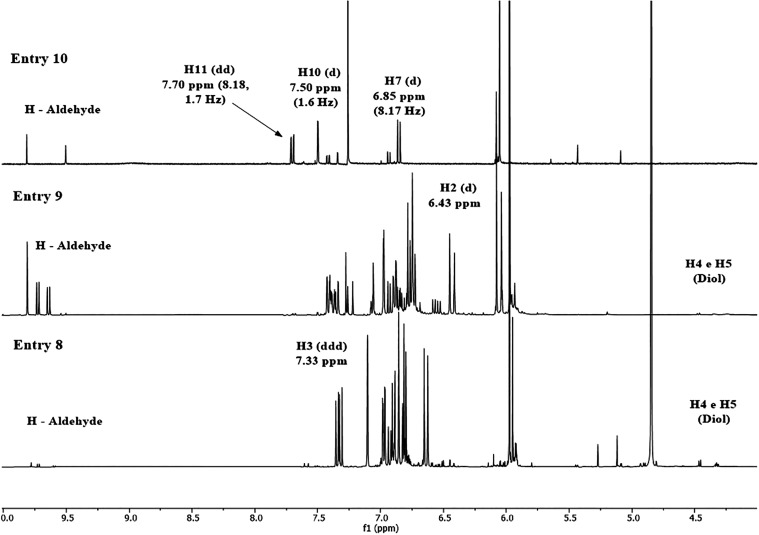
^1^H NMR spectra of solutions resulting from reactions carried out in the presence of Lewis acids under the conditions described in Table 2.

### Toxicity/Carcinogenicity and Anticarcinogenicity Studies

The experiments were performed in vivo in *D. melanogaster* using commercial mineral oil as a solvent for the treatments due to the low solubility of PIP in distilled water. No significant differences in toxicity or carcinogenicity were observed between distilled water (solvent control; SC) and mineral oil (negative control; NC). Therefore, SC data were omitted from the tables and figures. Doxorubicin (DXR, 0.4 mM) was used as a positive control (PC) because it induces point mutations and homologous recombination in *D. melanogaster* in the Epithelial Tumor Test (ETT).^36^

The survival rate of larvae treated with PIP alone or PIP + DXR was greater than 80% in all treatments, with no significant differences relative to the NC or PC. Similar results were observed for larvae treated with 4,5-dihydroxypiperine (dhPIP) or dhPIP + DXR (Figure 7). A survival rate of at least 70% is considered ideal and nontoxic for *D. melanogaster*.^37–39^

**Figure 7:**
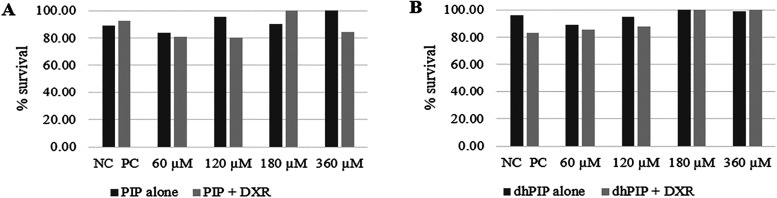
Survival of *D. melanogaster* evaluated after metamorphosis from third-stage larvae. (A) Larvae were treated with different concentrations of piperine (PIP) alone or in combination with doxorubicin (PIP + DXR). (B) Larvae were treated with different concentrations of 4,5-dihydroxypiperine (dhPIP) alone or in combination with doxorubicin (dhPIP + DXR). NC: mineral oil (negative control); PC: DXR 0.4 mM (positive control). No statistically significant differences (*p* < 0.05) were found between treatments and controls according to ANOVA.

The Epithelial Tumor Test (ETT) is based on monitoring epithelial tumor formation in adult fly bodies. In wild-type *D. melanogaster*, the *wts* marker is a tumor suppressor gene. Epithelial tumors arise from the loss of heterozygosity at the *wts* locus, which occurs when heterozygous diploid cells lose the *wts* marker, allowing the formation of invasive circular cell clones, known as warts.^40^ This process occurs mainly through chromosomal deletions or mitotic recombination and, to a lesser extent, through segregation defects.^41^ In the ETT, only flies with the (*wts* + /+ *mwh*) genotype were evaluated for epithelial tumors. Tumor occurrence was highest in flies treated with DXR alone during the larval stage. The tumors were primarily concentrated in the body, wings, and head (Figure 8). The greater incidence of tumors in these regions can be attributed to the higher number of cells present compared to other appendages.^42^

**Figure 8:**
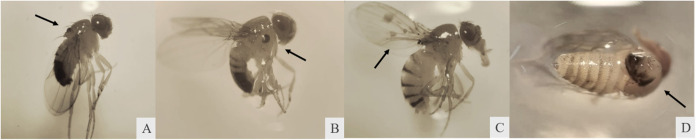
Epithelial tumors observed in different parts of the body of *D. melanogaster* treated with DXR (0.4 mM). (A), (B) and (D) tumors in the body; (C) tumors in the wings.

PIP alone did not induce mutagenic or genotoxic damage leading to loss of heterozygosity at the *wts* locus, as no significant increase in tumor frequency was observed in flies treated with PIP at different concentrations (Table 3). A genotoxic effect at similar concentrations of PIP has been reported in the *Allium cepa* bioassay, associated with an increase in micronuclei and nuclear buds, both of which are indicators of mutagenic alterations.^43^ Interestingly, a lower tumor frequency was observed at 120 μM, suggesting a protective effect against spontaneous DNA damage.

**Table 3:** Frequency of Tumors in *D. melanogaster* Descendants Heterozygous for the *wts* Gene, Treated with Various Concentrations of PIP Alone, Co-Treated with DXR, or Respective Controls^a^,^b^,^c^ ^d^

treatments			number of tumors analyzed								
DXR (mM)	PIP (μM)	N	eye	had	wing	body	leg	hater	total	%T^c^	**%**I^d^
0	0	175	0	1	4	8	1	0	14	8.0	
0	60	241	0	2	1	10	0	0	13	5.4	
0	120	211	0	0	0	3	0	0	3	1.4	
0	180	171	0	1	0	10	0	0	11	6.4	
0	360	183	1	0	2	4	0	1	8	4.4	
0.4	0	260	0	10	12	36	0	1	59	22.7*	
0.4	60	230	0	0	3	9	0	0	12	5.2#	77.0
0.4	120	194	0	3	4	7	0	0	14	7.2#	68.2
0.4	180	190	1	0	2	4	1	0	8	4.2#	81.4
0.4	360	208	2	1	3	9	1	0	16	7.7#	66.1

c Statistical diagnoses according to the chi-square test for independent samples. Significance level (*p* = 0.05).

d *, # Values considered different from the negative and positive controls, respectively. *N*: number of flies.

a Tumor frequency: (number of flies with tumors/number of flies evaluated) × 100.

b DXR-induced damage reduction index: (% tumors in DXR alone treatment – % tumors in PIP cotreatments)/% tumors in DXR alone treatment.

In the PIP + DXR cotreatment, a decrease in tumor frequency was observed across different concentrations (Table 3). PIP modulated the carcinogenic activity of DXR, inhibiting tumor formation by up to 81% at 180 μM.

DhPIP was noncarcinogenic at all tested concentrations (Table 4). All evaluated dhPIP concentrations modulated the carcinogenic activity of DXR, reducing the DNA damage it induced. The number of tumors per fly was reduced by up to 83% at 60 μM.

**Table 4:** Frequency of Tumors in *D. melanogaster* Descendants Heterozygous for the *wts* Gene, Treated with Various Concentrations of dhPIP Alone, Co-Treated with DXR, or Respective Controls^a^,^b^,^c^,^d^

treatments			number of tumors analyzed								
DXR (mM)	dhPIP (μM)	N	eye	had	wing	body	leg	hater	total	% T^c^	% I^d^
0	0	134	0	0	0	3	0	0	3	2.2	-
0	60	136	0	0	1	1	0	0	2	1.5	-
0	120	128	0	0	2	2	0	0	4	3.1	-
0	180	151	0	0	2	2	0	0	4	2.7	-
0	360	149	1	0	0	1	0	0	2	1.3	-
0.4	0	163	0	2	11	13	0	0	26	16.0*	-
0.4	60	148	0	1	2	1	0	0	4	2.7#	83.1
0.4	120	206	1	0	5	5	0	0	11	5.3#	66.5
0.4	180	164	0	4	2	2	0	0	8	4.9#	69.4
0.4	360	161	0	1	5	2	0	0	8	5.0#	68.8

c Statistical diagnoses according to the chi-square test for independent samples. Significance level (p = 0.05).

d *, # Values considered different from the negative and positive control, respectively. *N*: number of flies.

a Tumor frequency: (number of flies with tumors/number of flies evaluated) × 100.

b DXR-induced damage reduction index: (% tumors in DXR alone treatment – % tumors in dhPIP cotreatments)/% tumors in DXR alone treatment.

## Conclusions

The catalytic activities of AD-mix-α and RuCl_3_/NaIO_4_ were evaluated in the asymmetric dihydroxylation of PIP olefins under various reaction conditions. The purified products were characterized by ^1^H and ^13^C NMR. For AD-mix-α, the optimal conditions were 25 °C, with similar results observed without 1 equiv of MSA. Under these conditions, approximately 51% of the product was obtained. The effect of MSA became more pronounced at lower catalyst loadings and reduced temperatures. The optimized process achieved yields superior to those reported in previous studies.

Although dhPIP mediated by RuCl_3_/NaIO_4_ was less efficient than the AD-mix-α protocol, it showed potential for methodological optimization by adjusting the reaction variables explored in this study.

PIP and dhPIP were nontoxic to *D. melanogaster* at the tested concentrations. In the Epithelial Tumor Test, neither compound exhibited mutagenic or carcinogenic potential. Furthermore, both PIP and dhPIP modulated the mutagenic and carcinogenic effects of DXR, inhibiting tumor formation at all evaluated cotreatment concentrations, particularly at 180 μM for PIP (81% reduction) and 60 μM for dhPIP (83% reduction).

## Methods

### Materials

Piperine, AD-mix-α, methanesulfonamide (MSA; CH_5_NO_2_S), sodium sulfite (Na_2_SO_3_), anhydrous sodium sulfate (Na_2_SO_4_), sodium metaperiodate (NaIO_4_), ruthenium chloride (RuCl_3_), sodium bicarbonate (NaHCO_3_), sodium thiosulfate (Na_2_S_2_O_3_), sulfuric acid (H_2_SO_4_), iron chloride (FeCl_2_), zinc chloride (ZnCl_2_), cerium sulfate (Ce_2_(SO_4_)_3_), and deuterated solvents were acquired from Sigma-Aldrich and used as received. tert-Butanol (tBuOH), chloroform, acetone, ethyl acetate (EtOAc), and acetonitrile (ACN) were HPLC-grade from Panreac and used without further purification. Doxorubicin hydrochloride (DXR; 0.4 mM) was obtained as Doxolem from Eurofarma Laboratórios Ltd.a. Instant mashed potato was purchased commercially under the Yoki brand.

### Instrumentation

Mass analyses were performed on a Bruker AutoFlex Max MALDI-TOF-MS spectrometer using an α-cyano-4-hydroxycinnamic acid (HCCA) matrix solubilized in chloroform. ^1^H and ^13^C NMR analyses were carried out on a 500 MHz Agilent Technologies 500/54 Premium Shielded spectrometer, using CDCl_3_ or CD_3_OD as solvents at 26.0 °C. The enantioselectivity of the reaction was determined by high-performance liquid chromatography (HPLC) using a chiral stationary phase on Shimadzu Prominence LC system. The analysis was performed with a 20 μL injection volume and diode array detection (DAD) at 254 nm. A Chiralpak IB chiral column (Daicel), with a particle size of 5 μm and dimensions of 4.6 × 250 mm^2^, was employed. The mobile phase consisted of an isopropanol/hexane gradient (5–100%), at a flow rate of 0.4 mL.min^–1^, a column temperature of 25 °C, and a total run time of 45 min. Circular dichroism (CD) spectra were recorded on a Jasco J-815 spectropolarimeter using standard sensitivity (100 mdeg). Measurements were performed in methanol, and spectra were collected in the range of 190–400 nm with a data pitch of 0.05 nm in continuous scanning mode at a scan speed of 20 nm.min^–1^, response time of 2 s, and bandwidth of 1 nm. Each spectrum corresponds to the average of two scans.

### Asymmetric Dihydroxylation

#### Method 1

AD-mix-α (500 mg) was dissolved in 10 mL of a *t*-BuOH/H_2_O solution (1:1 v/v) in a round-bottom flask at room temperature. Piperine (100 mg) and methanesulfonamide (MSA, 33 mg) were then added, and the mixture was allowed to react for 24 h at 25 °C. Sodium sulfite (0.90 g) was added, and the reaction was stirred for an additional 2 h. Then, 25 mL of distilled water was added. The mixture was extracted with EtOAc (3 × 25 mL^2^), and the combined organic phases were dried over anhydrous Na_2_SO_4_. The solvent was evaporated, and the residue was purified on a silica gel column using a chloroform/methanol eluent of increasing polarity. The product was characterized by ^1^H and ^13^C NMR and mass spectrometry.

#### Method 2

NaIO_4_ (107 mg) was dissolved in 0.25 mL of water in a round-bottom flask at room temperature, followed by the addition of 66 mL of 1 M H_2_SO_4_ and 10 mol % of FeCl_2_, ZnCl_2_, or Ce_2_(SO_4_)_3_. After complete dissolution of the solids, the solution was cooled to 0 °C, and 16 mL of 0.1 M RuCl_3_ was added. Ethyl acetate/dichloromethane/acetone (1 mL) was added and stirred for 5 min. Then, 1 mL of acetonitrile was added, and stirring continued for an additional 5 min. Piperine (95 mg) was then added, and the resulting solution was stirred at 0 °C for 5–120 min. To terminate the reaction, 2.5 mL of saturated NaHCO_3_ and 3.4 mL of saturated Na_2_S_2_O_3_ solutions were added. The phases were separated, and the aqueous phase was extracted with ethyl acetate (3 × 10 mL^2^). The combined organic phases were dried over Na_2_SO_4_, and the solvent was evaporated. The residue was dissolved in 650 μL of CD_3_OD containing an internal standard of 2.2 × 10^–3^ M TMS. The yield/purity (P_Sample_) was determined by quantitative NMR (qNMR) measurements^44^ according to the following equation

$$P_{\mathrm{Sample}}=\frac{I_{\mathrm{Analyte}}}{I_{\mathrm{CRM}}}\times \frac{N_{\mathrm{CRM}}}{N_{\mathrm{Analyte}}}\times \frac{M_{\mathrm{Analyte}}}{M_{\mathrm{CRM}}}\times \frac{m_{\mathrm{CRM}}}{m_{\mathrm{Sample}}}\times P_{\mathrm{CRM}}$$

where P_Sample_ is the purity of the sample as a mass fraction; P_TMS_ is the purity of the TMS as a mass fraction; I_Analyte_ is the integral of the analyte signal; I_TMS_ is the integral of the TMS signal; N_Analyte_ is the number of hydrogens in the analyte signal; N_TMS_ is the number of hydrogens in the TMS signal; M_Analyte_ is the molar mass of the analyte; M_TMS_ is the molar mass of the TMS; m_Sample_ is the mass of the sample; m_TMS_ is the mass of the TMS.

### Piperine and 4,5-Dihydroxypiperine Characterizations

#### Piperine (PIP; Yellow Solid)

^1^H NMR (400 MHz, CDCl_3_) δ 7.43 (1H, ddd, j = 14.7, 9.4, 0.9 Hz), δ 6.98 (1H, d, j = 1.7 Hz), δ 6.89 (1H, dd, j = 8.1, 1.7 Hz), δ 6.76 (3H, m), δ 6.43 (1H, d, j = 14.7 Hz), δ 5.97 (2H, s), δ 3.59 (4H, t), δ 1.68 (2H, m), δ 1.60 (4H, m); ^13^C NMR (126 MHz, CDCL_3_) δ 165.43, 148.21, 148.11, 142.46, 138.20, 131.03, 125.37, 122.50, 120.08, 108.51, 105.67, 101.29, 46.91, 43.23, 26.74, 25.63, 24.67.

#### 4*R*,5*R*-Dihydroxypiperine (dhPIP; White Solid)

^1^H NMR (500 MHz, CD_3_OD), δ 6.88 (1H, d, J = 1.6 Hz), 6.81 (1H, dd, J = 8.0, 1.6 Hz), 6.76 (1H, d, J = 7.9 Hz), 6.52 (1H, dd, J = 15.3, 4.9 Hz), 6.43 (1H, dd, J = 15.4, 1.4 Hz), 5.92 (2H, dd), 4.45 (1H, d, J = 6.7 Hz), 4.32 (1H, ddd, J = 6.6, 4.9, 1.5 Hz), 3.53 (2H, m), 3.41 (2H, t), 1.67 (2H, m), 1.52 (4H, m); ^13^C NMR (126 MHz, CD_3_OD) δ 166.03, 147.58, 147.14, 143.51, 135.10, 121.12, 120.42, 107.35, 107.15, 100.89, 76.71, 75.50, 46.77, 42.88, 26.33, 25.33, 24.03. CD (CH_3_OH, Δε [mdeg], λ [nm]) 206 (−31.84), 214 (−23.64), 282 (−5.10).

### Toxicity and Epithelial Tumor Test in *D. melanogaster* (ETT)

The ethics approval was not required for this study. Only studies with animals of species classified as phylum Chordata, subphylum Vertebrata require approval by the institutional ethics committee (Law No. 11,794, of October 2008).

The larvae of *D. melanogaster* were exposed to treatments containing PIP alone or PIP + DXR to determine the survival rate. Yoki instant mashed potato (2 g) was mixed with 1 mL of PIP in pure mineral oil at concentrations of 60, 120, 180, and 360 μM and distributed into eight vials. In half of the vials, 5 mL of distilled water was added for the mutagenesis assays, while in the other half, 5 mL of DXR (0.04 mM) was added to evaluate the modulatory effect on DNA damage.

Once the vials were prepared, 800 third-instar larvae from the cross between virgin *wts/TM3*, *Sb* ^*1*^ females and *mwh/mwh* males were counted and distributed into the eight vials (100 larvae per vial). Additional vials containing only mashed potato or mashed potato mixed with mineral oil were also moistened with distilled water or DXR (0.4 mM) and used as solvent (SC), negative (NC), and positive (PC) controls, with 100 larvae per vial.

The hatched flies were counted and stored in 70% ethanol. The experiment was performed in triplicate, allowing comparisons between treatments and their respective controls using ANOVA at a significance level of *p* < 0.05, with analyses conducted in the statistical program Bioestat 5.0. Similarly, a second assay was performed to evaluate the survival rate of larvae exposed to the same concentrations of dhPIP alone or dhPIP + DXR.

After evaluating the survival rate for both assays, the phenotype of the progeny was evaluated under a stereomicroscope to separate the two types of descendants: (I) *mwh* + /+ *TM3, Sb* ^*1*^; (II) *mwh* + /+ *wts*, which can be differentiated phenotypically due to the presence of the chromosomal balancer *Sb* ^*1*^, which confers short and thick hairs to the flies.^45^

Only individuals carrying the gene of interest (*wts*) were evaluated for the occurrence of epithelial tumors. The number of tumors and their positions on the fly’s body were recorded. Tumor frequencies in the treatments and their respective controls were compared using the chi-square test for independence at a significance level of 5%.

The frequency of tumors per 100 flies was calculated as Frequency of tumors per 100 flies = 100­(Number of flies evaluated)/(Number of tumors).

The DXR-induced damage reduction index for the cotreatments was calculated using the following equation: Damage reduction (%) = [(% tumors in DXR treatment) – % tumors in (PIP or dhPIP + DXR treatment)]/(% tumors in DXR treatment).

## Supplementary Material



## Data Availability

The data underlying this study are available in the published article and its Supporting Information.

## Notes

Tables detailing the reaction conditions for experiments conducted at 15 °C; NMR signal assignments for the dhPIP molecule; MALDI-TOF spectra of the asymmetric dihydroxylation syntheses; and the ^1^H NMR spectra of the RuCl_3_/NaIO_4_-mediated syntheses; the table and chromatogram of the chiral HPLC analysis; and the circular dichroism (CD) spectrum are available as a freely accessible (PDF)
