# Supplementary material for: The Anticarcinogenicity Effects of Piperine and 4,5-Dihydroxypiperine in Drosophila melanogaster Somatic Cells
Source: ACS Omega. 2026 Mar 30;11(14):22072–81. doi: 10.1021/acsomega.5c13279 (PMC13084445; doi:10.1021/acsomega.5c13279)
Supplement: Supplementary file 1 [file ao5c13279_si_001.pdf]

SUPPLEMENTARY MATERIAL

The anticarcinogenicity effects of piperine and 4,5-dihydroxypiperine in *Drosophila melanogaster* somatic cells

Wellington S. Aguiar[a], Isabelle V. A. Pires[b], Lívia M. L. Barros[b], Pedro M. Almeida[a,b], José L. S. Sá[a], Benedito S. Lima-Neto[c], Francielle A. Martins[a,b*]

[a]Graduate Program in Chemistry, State University of Piauí, Teresina, Piauí, Brazil;

[b]Genetics Laboratory, Center of Natural Sciences, State University of Piauí, Teresina, Piauí, Brazil;

[c]Institute of Chemistry of São Carlos, University of São Paulo, São Carlos, SP, Brazil.

Table S1

m/z signals of 4,5-dihydroxypiperine (dhPIP) ions observed by MALDI-TOF, obtained from syntheses using AD-mix-α in *t*-BuOH/H_2O (1:1) for 24 h, with varying percentages of oxidant and methanesulfonamide (MSA).

Entry	Temperature (°C)	Oxidant (mol %)	MSA	m/z observed
7	15	0.4	1 eq	302, 320
8	15	0.4	3 eq	302, 320, 342
9	15	0.8	3 eq	302, 320, 342

Table S2

^{1}H and ^{13}C NMR data (CD_3OD) of 4,5-dihydroxypiperine (dhPIP) obtained using AD-mix-α in *t*-BuOH/H_2O (1:1) under the conditions of Entry 8.

Position	δ_C, type	δ_H (multiplet, *J*, Hz)
**1**	166.03, C	
**2**	121.12, CH	6.43 (dd, 15.4, 1.4)
**3**	143.53, CH	6.52 (dd, 15.3, 4.9)
**4**	75.50, CH	4.32 (ddd, 6.6, 4.9, 1.5)
**5**	76.71, CH	4.45 (d, 6.7)
**6**	135.10, C	
**7**	107.35, CH	6.76 (d, 7.9)
**8**	147.58, C	
**9**	147.14, C	
**10**	107.15, CH	6.88 (d, 1.6)
**11**	120.42, CH	6.81 (dd, 8.0, 1.6)
**12**	100.89, CH_2	5.92 (dd)
**1'**	42.88, CH_2	3.41 (t)
**2'**	26.33, CH_2	1.52 (m)
**3'**	24.03, CH_2	1.67 (m)
**4'**	25.33, CH_2	1.52 (m)
**5'**	46.77, CH_2	3.53 (m)

Table S3

Chromatographic parameters and peak area integration for 4,5-dihydroxypiperine obtained by chiral HPLC using a Chiralpak IB column (Daicel) with an isopropanol/hexane gradient (5–100%), flow rate of 0.4 mL min^{-1}, column temperature of 25 °C, and total analysis time of 45 min.

**Peak**	**Ret. Time**	**Area**	**Height**	**Area%**	**Height%**
1	26.051	30786186	1195625	99.450	99.676
2	29.968	170223	3887	0.550	0.324
**Total**		30956409	1199512	100.000	100.000

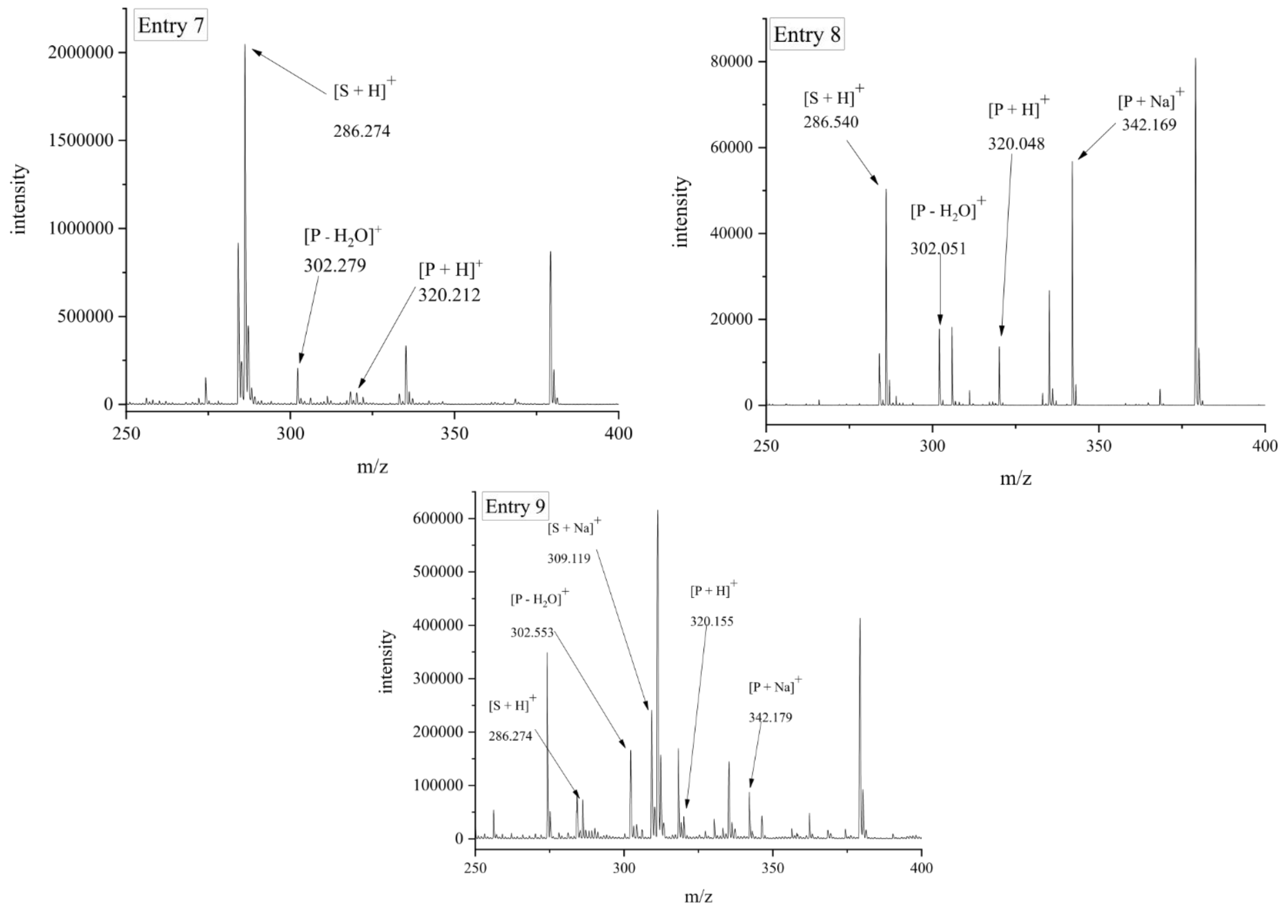

Figure S1. MALDI-TOF spectra of asymmetric dihydroxylation syntheses using AD-mix-α in *t*-BuOH/H_2O (1:1) at 15 °C for 24 h, corresponding to Entries 7, 8, and 9 (Table S1). The m/z signals corresponding to piperine (PIP) are labeled "S," and those corresponding to 4,5-dihydroxypiperine (dhPIP) are labeled "P."

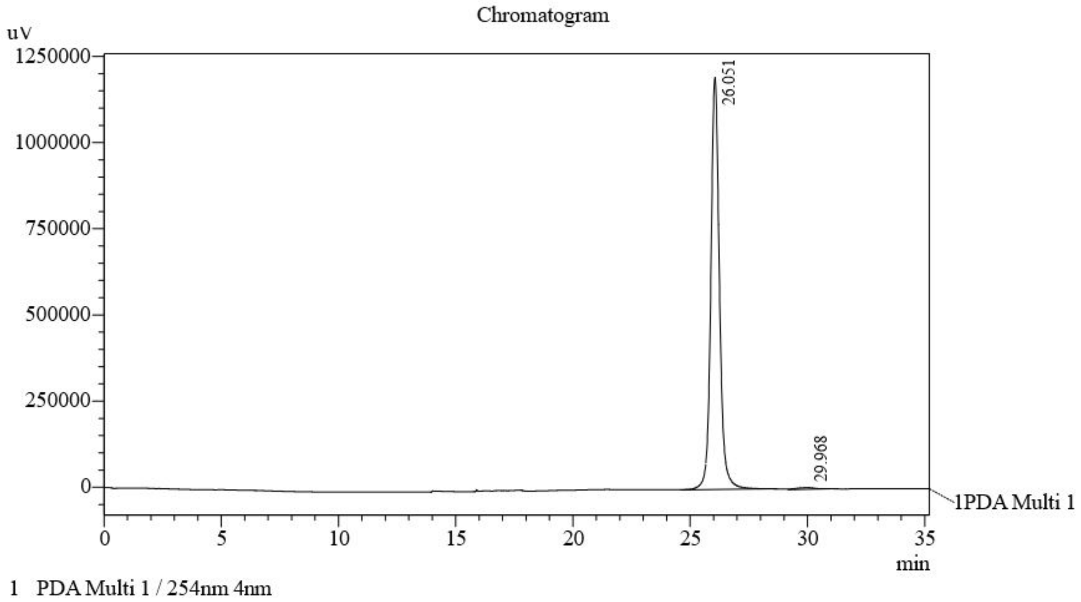

Figure S2. Chromatogram for 4,5-dihydroxypiperine obtained by chiral HPLC using a Chiralpak IB column (Daicel) with an isopropanol/hexane gradient (5-100%), flow rate of 0.4 mL min^{-1}, column temperature of 25 °C, and total analysis time of 45 min.

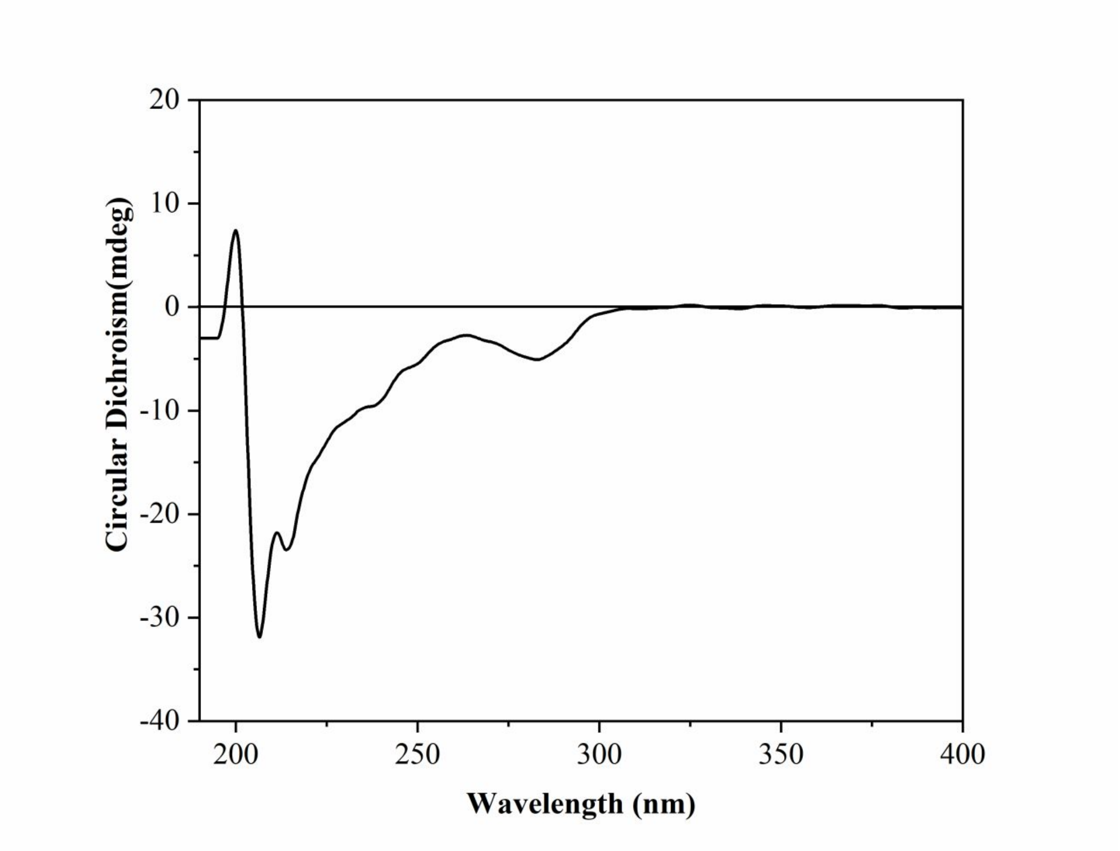

Figure S3. Circular dichroism spectrum of 4,5-dihydroxypiperine in methanol.

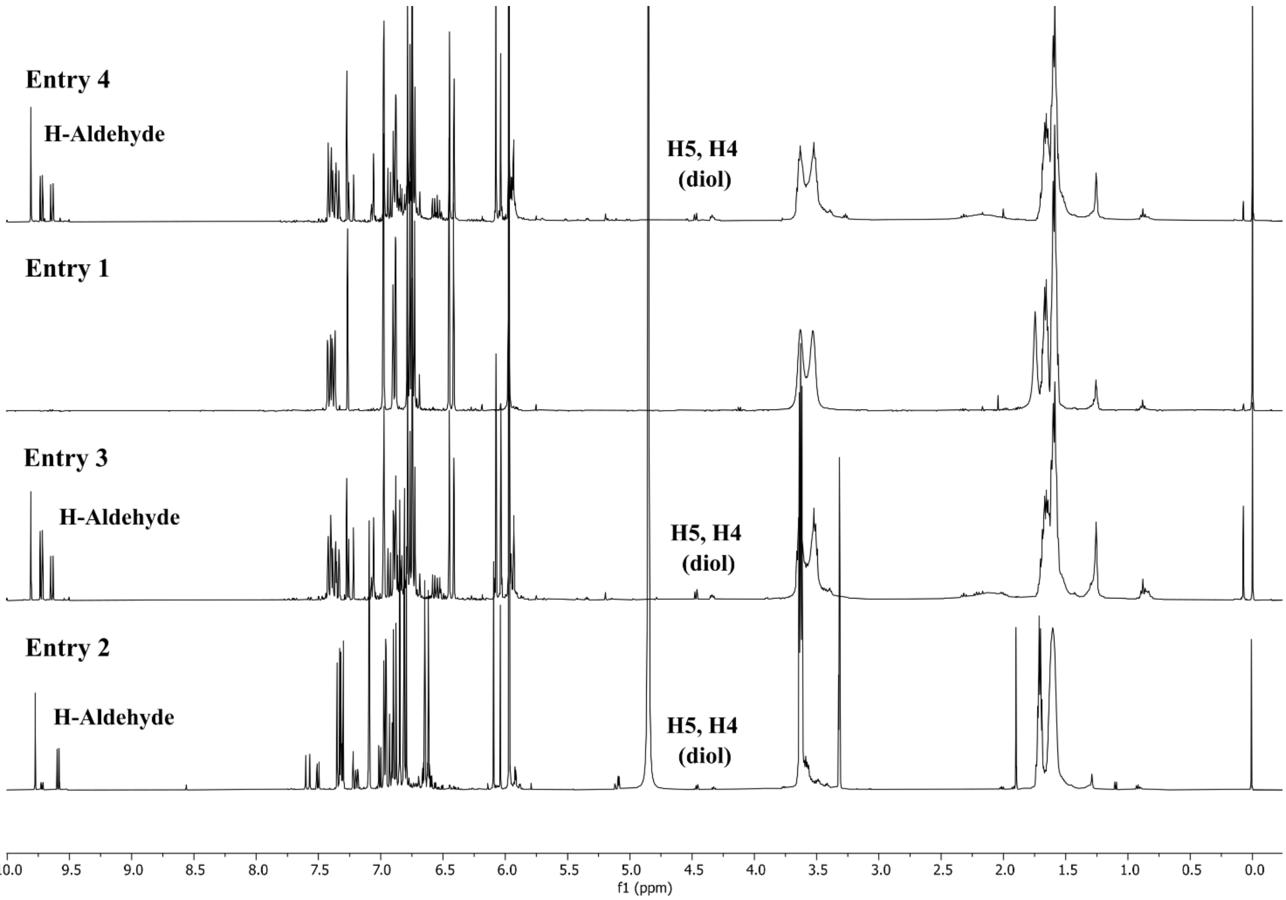

Figure S4. ^{1}H NMR spectra of dhPIP from syntheses performed without acids (Table 2), showing diol hydrogen signals in the 4.0–4.5 ppm region.

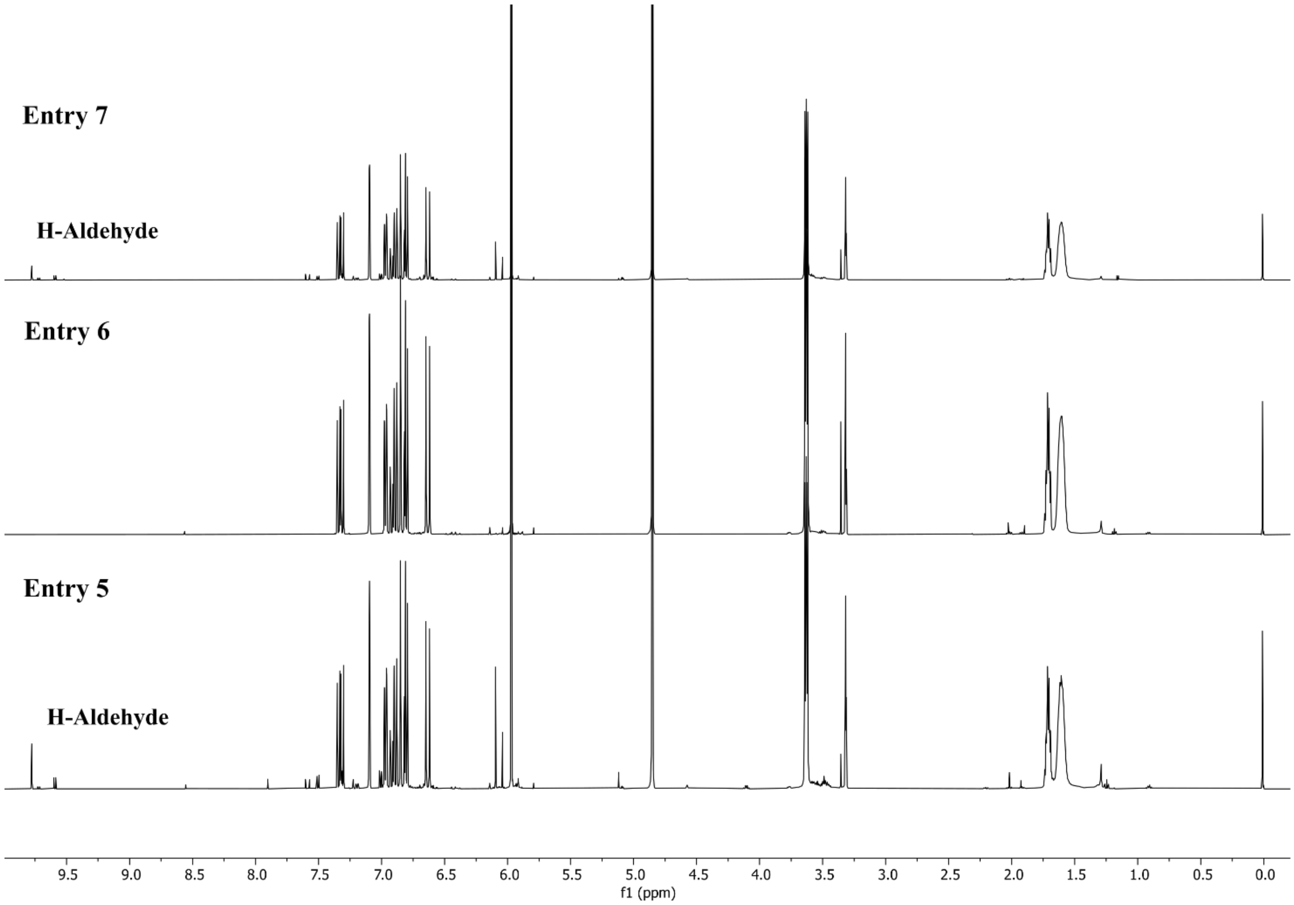

Figure S5. ^{1}H NMR spectra of dhPIP obtained from syntheses performed in the presence of Brønsted acids (Table 2). No hydrogen signals corresponding to the diol moiety were observed in the region of 4.0–4.5 ppm.
